# ViralLink: An integrated workflow to investigate the effect of SARS-CoV-2 on intracellular signalling and regulatory pathways

**DOI:** 10.1371/journal.pcbi.1008685

**Published:** 2021-02-03

**Authors:** Agatha Treveil, Balazs Bohar, Padhmanand Sudhakar, Lejla Gul, Luca Csabai, Marton Olbei, Martina Poletti, Matthew Madgwick, Tahila Andrighetti, Isabelle Hautefort, Dezso Modos, Tamas Korcsmaros

**Affiliations:** 1 Earlham Institute, Norwich, United Kingdom; 2 Quadram Institute Bioscience, Norwich, United Kingdom; 3 Department of Genetics, Eotvos Lorand University, Budapest, Hungary; 4 Department of Chronic Diseases, Metabolism and Ageing, Translational Research Center for Gastrointestinal Disorders, Katholieke Universiteit Leuven, Leuven, Belgium; 5 Institute of Biosciences, São Paulo University, Botucatu, Brazil; bioinformatics, GERMANY

## Abstract

The SARS-CoV-2 pandemic of 2020 has mobilised scientists around the globe to research all aspects of the coronavirus virus and its infection. For fruitful and rapid investigation of viral pathomechanisms, a collaborative and interdisciplinary approach is required. Therefore, we have developed ViralLink: a systems biology workflow which reconstructs and analyses networks representing the effect of viruses on intracellular signalling. These networks trace the flow of signal from intracellular viral proteins through their human binding proteins and downstream signalling pathways, ending with transcription factors regulating genes differentially expressed upon viral exposure. In this way, the workflow provides a mechanistic insight from previously identified knowledge of virally infected cells. By default, the workflow is set up to analyse the intracellular effects of SARS-CoV-2, requiring only transcriptomics counts data as input from the user: thus, encouraging and enabling rapid multidisciplinary research. However, the wide-ranging applicability and modularity of the workflow facilitates customisation of viral context, *a priori* interactions and analysis methods. Through a case study of SARS-CoV-2 infected bronchial/tracheal epithelial cells, we evidence the functionality of the workflow and its ability to identify key pathways and proteins in the cellular response to infection. The application of ViralLink to different viral infections in a context specific manner using different available transcriptomics datasets will uncover key mechanisms in viral pathogenesis.

This is a *PLOS Computational Biology* Software paper.

## Introduction

By the end of June 2020 roughly 23,500 scientific publications were released relating to Severe Acute Respiratory Syndrome Coronavirus 2 (SARS-CoV-2) and the disease it causes (COVID-19) [1]. This fast uptake in research efforts is vital to decrease the health and economic impacts of this new pandemic. However, many questions remain unanswered regarding the molecular processes driving host responses to this coronavirus. One key challenge to utilisation of new findings is that published datasets are mostly unlinked to each other (due to parallel efforts by multiple research groups) and not always connected to community standard resources. An integrated and reusable method to interactively capture new data and connect it to existing data sources is needed. Such a comprehensive approach that can be run regularly when relevant new data is available, will increase and update our understanding of the mechanistic details of the SARS-CoV-2 infection. Further, it will aid drug target discovery by enabling identification of high confidence mediators through which the virus is affecting host cells [2]. Studying the effect of the virus at molecular level may explain the variety of clinical manifestations of the infection and the differences in susceptibility between different populations, and together with soon available human genomics data, could be used for identifying risk factors.

Upon entry of a virus into a human cell *via* surface receptors, viral RNA is released and translated into proteins [3]. In addition to their role in direct viral replication, these proteins are able to bind to human proteins creating a host-virus interface [4]. This interaction can lead to downstream signalling changes in the host cell, either as a result of viral hijacking or through a defined viral immune response by the host cell [5]. Ultimately, this signal flow results in intracellular gene transcription changes, cell-cell signalling and systemic host responses which drive the tug-of-war between the host and the virus [6]. In order to understand and control this conflict, it is necessary to study each of these levels of host response in detail, including the intracellular response of the primarily infected cell.

Currently available data relating to intracellular SARS-CoV-2 infection includes human binding partners of viral proteins [4,7,8] and transcriptomics datasets from infected cell lines [9–11], infected patients [12,13] and other infected animals [11,14]. Interdisciplinary and collaborative science can maximise the value of each of these datasets through data integration and comparison combined with application of different computational analysis approaches. One such computational analysis method is the utilisation of network approaches to model molecular interactions between the virus and human proteins as well as within and between human cells [15]. Network approaches have already been applied to study SARS-CoV-2 pathogenesis and to predict drug repurposing candidates and master regulators based on proteins in proximity to human binding proteins (which physically associate with SARS-CoV-2 proteins) [16–20].

Here we present a systems biology workflow built on our previously published resource MicrobioLink, which studies the effect of viral infections on host cells [21]. ViralLink reconstructs and analyses a causal molecular interaction network whose signal starts with the binding of an intracellular viral protein to a human protein, travels via multiple signalling pathways, and ends at the transcriptional regulation of altered genes. Subsequently, the workflow investigates the causal network using betweenness centrality measures, cluster analysis, functional overrepresentation analysis and network visualisation. Using currently available datasets from SARS-CoV-2 infected bronchial epithelial cells we demonstrate that this workflow can identify biologically relevant signalling pathways and predict key proteins for potential drug interventions. The workflow is built in a modular, standardised and updateable fashion and requires only limited programming ability to run. ViralLink can be applied easily to new SARS-CoV-2 related datasets or datasets from other viruses, to study the effect of the virus on host signalling and regulation in diverse contexts—including different cell types, patients and viral strains.

## Design and implementation

### ViralLink workflow overview

**Fig 1:**
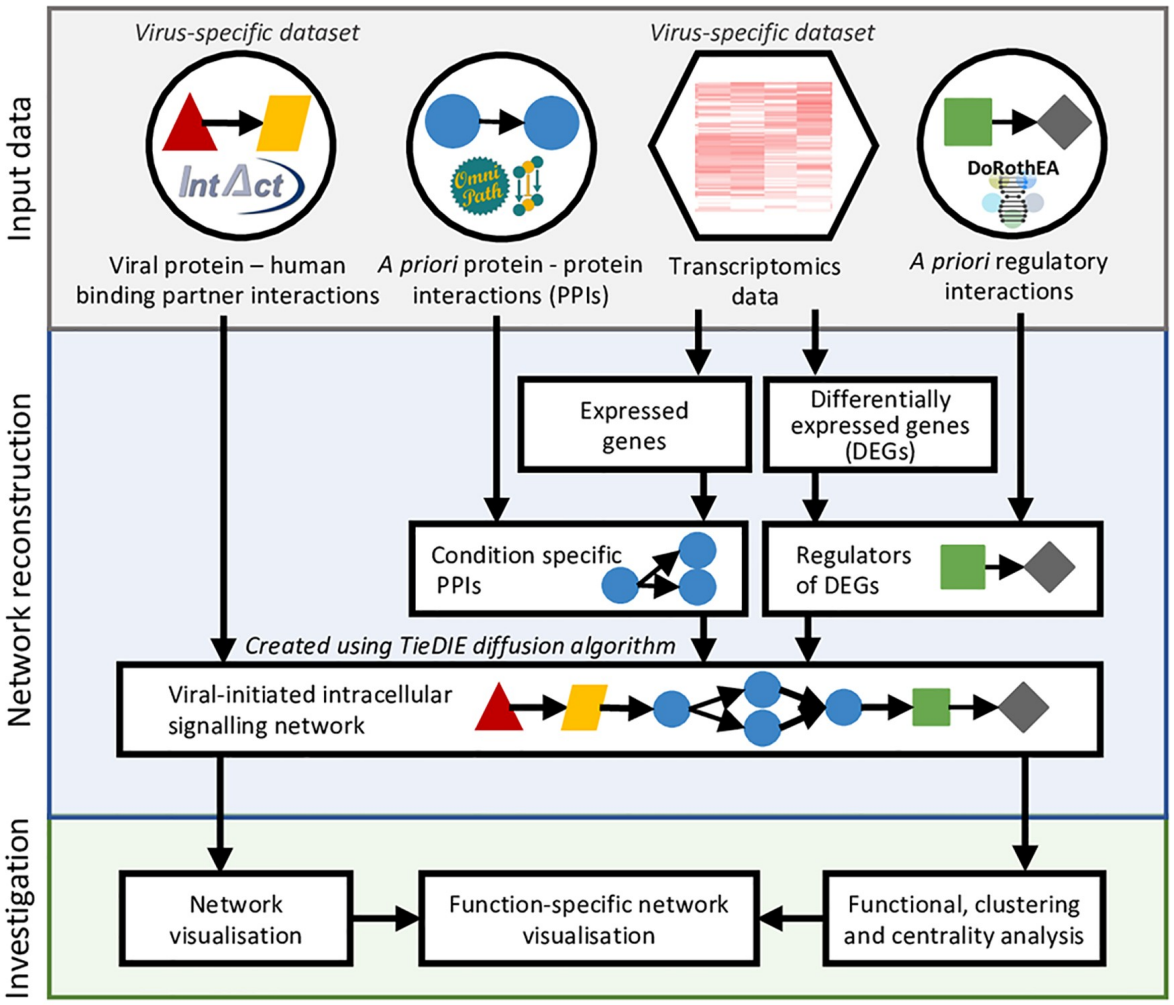
ViralLink workflow overview.

The ViralLink workflow investigates the effect of viral infection within cells by generating and analysing context-specific networks of intracellular signalling and regulatory molecular interactions. These networks link the intracellular binding of viral and human proteins to the transcriptional response of the infected cell (Fig 1). The context-specificity of the analysis is obtained through the choice of input transcriptomics datasets—it could refer to strain of virus, type of infected cell, severity of infection, age of host or any other context of interest. By default, the workflow is set up to analyse the intracellular effects of SARS-CoV-2, requiring only transcriptomics counts data as input and thus encouraging and enabling rapid multidisciplinary research. However, the wide-ranging applicability and modularity of the workflow facilitates customisation of viral context, *a priori* interactions and analysis methods. ViralLink contains three primary stages: 1) collection and input of data; 2) reconstruction of the network; and 3) investigation of results using functional analysis, clustering, centrality measures and visualisation.

### ViralLink workflow methods

#### Collection and input of data

Reconstruction of causal networks using ViralLink requires four separate input datasets (Fig 1): viral protein-human binding protein interactions, *a priori* human protein-protein interactions (PPIs), *a priori* human transcription factor (TF)—target gene (TG) interactions and an unnormalised counts matrix from a gene expression experiment. By default, all data except the transcriptomics counts are provided automatically. However alternative input files can be provided if desired.

The default workflow uses SARS-CoV-2 protein-human binding protein interactions obtained from IntAct [22,23]. This data was reformatted to contain one row per molecular interaction with 2 columns of UniProt IDs: SARS-CoV-2 proteins and human binding proteins. Alternative viral-human PPIs can be provided using the same data format. The workflow assumes all viral-human interactions have an inhibiting action on the human protein, unless a third column named “sign” is present in the input file containing “+” for activatory and “-” for inhibitory interactions. In addition, data is provided with the workflow containing the gene names corresponding to each of the SARS-CoV-2 proteins, to enable easy interpretation of the reconstructed networks.

For *a priori* human interactions, the workflow obtains and uses integrated collections of PPI and TF-TG interactions from OmniPath and DoRothEA, respectively [24,25]. These interactions are obtained using the ‘OmniPathR’ R package [24,26] to download and filter signed and directed interactions. For DoRothEA, only high and medium confidence level interactions are used (confidence scores A-C). In contrast to importing static input files, this script enables the use of up-to-date interaction data. Alternative interaction data can be used with the workflow provided it has the same format: specifically, it must contain source and target UniProt IDs in the columns ‘to’ and ‘from’ and if the transcriptomics data uses gene symbols, the interaction data must additionally contain gene symbols in the columns ‘source_genesymbol’ and ‘target_genesymbol’. Furthermore, the interactions must be directed and signed with the sign of the interaction given in the column ‘consensus_stimulation’ where the value ‘1’ represents a stimulation and anything else represents an inhibition.

The aforementioned *a priori* interactions are contextualised using transcriptomics data from any study of interest which compares viral infected to uninfected human cells or tissues. Correspondingly, the workflow requires unnormalised counts data from a transcriptomics experiment (containing UniProt or gene symbols as IDs) and a corresponding mapping table which lists the sample IDs (from the headers of the counts table) in the ‘sample_name’ column and the ‘test’ or ‘control’ status of the sample in the ‘condition’ column. This mapping table is used to carry out differential expression of a test condition (e.g. infected) compared to a control condition (e.g. uninfected). An example expression dataset and mapping table are provided with the workflow.

To process the transcriptomics data, the workflow uses ‘DESeq2’ in R to normalise the counts and to carry out differential expression analysis [27]. Any genes passing the log2 fold change and adjusted p value cut-offs, based on the provided parameters (default 1 and 0.05, respectively), are classed as differentially expressed genes (DEGs). Following removal of all genes with count = 0, normalised log2 counts across all samples are fitted to a gaussian kernel [28]. All genes with expression values above mean minus three standard deviations are considered as expressed genes. Subsequently, context-specific human PPI and TF-TG interactions are generated by filtering only interactions where both interacting molecules are expressed.

File paths to all input datasets and associated parameters (such as desired log2 fold change cut off) are specified in the parameters text file which is read in by the workflow.

#### Network reconstruction

The reconstructed causal network contains three layers of interactions, which are obtained, by default, from the three *a priori* interaction resources:

- Viral proteins interacting with human binding partners: from the SARS-CoV-2 collection in the IntAct database [22,23]
- Intermediary signalling protein interactions: from protein-protein interactions (PPIs) of the OmniPath collection [24]
- Transcription factors (TFs) regulating differentially expressed genes: from a transcriptomics dataset of interest and the DoRothEA collection [25]

A list of all TFs targeting the differentially expressed genes are obtained from the context-specific TF-TG interactions. The human binding proteins of viral proteins are connected to the listed TFs through the context-specific human PPIs using a network diffusion approach called Tied Diffusion Through Interacting Events (TieDIE) [29]. As inputs for the TieDIE tool, the following information is used: 1) The signed, directed and expression based filtered PPIs is used as the input network. 2) Human proteins which are interacting partners of the viral proteins are used as the start nodes. The number of viral proteins bound to each of the human proteins are assigned as the weights of the start nodes. 3) The TFs of the DEGs in the dataset are used as the stop nodes. The weights for each of the TFs in the set of stop nodes were calculated using the following formula (Eq 1) which considers both the log2 fold change of the DEGs as well as the sign (i.e. stimulatory or inhibitory) of the relationship between the TF and the DEG.

$$\begin{array}{ll} Weight_{TF}= & \frac{1}{N_{TFTG}}\sum_{n\in TG}LFC_{n}\cdot sign(TFTG_{n})\\ sign(x)= & \{\begin{array}{c} x=+1\;if\;x=activatory,\\ x=-1\;if\;x=inhibitory. \end{array} \end{array}$$ (1)

After running TieDIE, a custom R script is used to collate all the data into a final viral-initiated intracellular signalling network (causal network), outputting an edge table representation of the network, with a node table containing additional node annotations. Starting with the interactions output from TieDIE, viral protein-human binding protein interactions are added for each of the present human binding proteins. Similarly, TF-TG interactions (where the TG is a DEG) are added for each of the present TFs, creating a full network with three interaction types: SARS-CoV-2 protein-human binding protein, PPI and TF-DEG. All nodes of the network are added to a node table with annotations including heat values (output from TieDIE), Entrez IDs (obtained in R using the ‘org.Hs.eg.db’ package), gene symbols (obtained from UniProt [30]) and log2 fold change values from the differential expression analysis.

#### Network investigation

Following reconstruction of the causal network, ViralLink provides functionality to investigate the results using functional analysis, clustering, centrality measures and visualisation.

*Centrality measures*. To identify key molecules in the reconstructed network ViralLink uses a betweenness centrality measure—calculating the global importance of a node (in this case a protein) based on the number of shortest paths which pass through them when connecting all node pairs in the network [31]. Nodes with high betweenness centrality play a key role in transduction of signals through the network, and here represent proteins with biological importance in the cellular response to viral infection. Betweenness centrality is calculated for each node in the causal network using the R package ‘igraph’ and output as an annotation in the node table [32]. Alternative centrality measures are available using the ‘igraph’ package and can be integrated into the workflow by the user if required.

*Functional analysis*. To further investigate important cellular functions and signalling pathways directly affected by the virus of interest, ViralLink carries out functional overrepresentation analysis on different parts of the causal network:

1. The DEGs of the network
2. The upstream human proteins (including human binding proteins, intermediary signalling proteins and TFs)
3. Identified clusters (only those with ≥ 15 nodes are investigated)

Functional overrepresentation analysis is carried out in R using packages ‘ClusterProfiler’ (for Gene Ontology annotations [33]) and ‘ReactomePA’ (for Reactome annotations [34–36]. For analysis of the upstream human signalling proteins and analysis of clusters, all proteins in the context-specific human PPI interactions are used as the background. For analysis of the DEGs, all target genes in the context-specific human TF-TG interactions are used as the background. For Gene Ontology (Biological Process) analysis (except when running the ‘compareCluster’ command), the ‘simplify’ command is used (select_fun = min) to remove redundant functions. All functions with q value ≤ 0.05 are considered significantly overrepresented.

Furthermore, ViralLink also implements a network-based pathway enrichment analysis on the upstream human proteins of the network using the ANUBIX (Adaptive NUll distriButIon of X-talk) algorithm [37]. Here, limitations of overrepresentation analysis, such as the assumption of gene independency, are overcome by integrating knowledge of gene/protein associations using networks. Specifically, upstream human proteins of the reconstructed causal network are tested for enrichment of ANUBIX-provided Reactome and KEGG pathways using the context-specific human PPI interactions as the input network. All functions with q value ≤ 0.05 are considered significantly overrepresented.

An additional R script is provided alongside the workflow which creates subnetworks of the causal network based on functions of interest. These function-specific subnetworks highlight how specific signalling pathways in the infected cell reach (and subsequently affect) specific functions of the DEGs. For example, the subnetwork could be created to show how viral proteins can affect different host toll-like receptor pathways, and how these pathways can ultimately affect DEGs associated with interleukins. In this network the DEG nodes would be replaced with nodes representing the interleukin functions (which must be overrepresented based on the functional analysis). This script requires the output files from the functional overrepresentation analysis, the node and edge tables of the causal network and a file of all UniProt IDs associated with all Reactome functions (which is provided with ViralLink, following download from the Reactome website in April 2020). In addition, the script requires a list of overrepresented DEG functions (Reactome) and a list of upstream signalling functions (Reactome) to visualise. The script outputs an edge table, a node table and a Cytoscape file.

*Visualisation*. Data visualisation is often an important part of biological network interpretation, providing new insights into the data and visually conveying analysis results [38]. As such, ViralLink has the capability to import reconstructed networks into the open-source Cytoscape network visualisation software [39,40]. Specifically, the workflow employs the ‘RCy3’ R package to interact with Cytoscape programmatically, importing the node and edge tables to create network visualisations and saving the data as a ‘.cys’ file. The causal network, the network clusters (where containing ≥ 15 nodes) and the function-specific networks are visualised in this way. If calculated previously, the causal network nodes are coloured based on their betweenness centrality, however further style and layout customisation must be carried out by the user directly based on the data.

*Cluster analysis*. Clustering algorithms are commonly used in network biology to investigate the complex structure of molecular interaction networks by extracting groups of densely connected molecules [41,42]. Depending on the number of molecules included, a cluster can represent a molecular complex or a group of molecules which function closely with each other. Cluster analysis can identify subsets of a large network with specific functions and indicate molecules that may have functional redundancy with each other—potentially having implications for drug targeting. ViralLink employs the MCODE clustering method to identify groups of densely connected nodes in PPI networks [41]. To carry out this analysis, ViralLink requires the Cytoscape software [39,40], which is controlled programmatically using the R package ‘RCy3’ with the Cytoscape ‘MCODE’ app (v1.6.1) [43]. MCODE is run using default parameters: degree cut off = 2, haircut = TRUE, node score cut off = 0.2, k-core = 2, max depth = 100. This analysis outputs the data as node annotations in the node table, which are used for the functional analysis and visualisation steps of the workflow.

### Implementation

The workflow consists of modular R and Python scripts which can be run in three separate ways: through a Docker container, through the provided Python wrapper script or as separate scripts for each step of the workflow. The Docker implementation is recommended to users as it does not require installation of R or Python or any of the necessary packages. The Python wrapper script and separate scripts are provided for more advanced users to enable bespoke analyses. If running ViralLink for the study of SARS-CoV-2, the only required input files are related to the transcriptomics data of interest: a raw counts table (using gene symbols or UniProt protein IDs) and a two-column metadata table specifying test and control sample IDs. One further script is provided to generate function-specific networks. This script is not included in the Docker container or Python wrapper because it requires the user to specify functions of interest from the output of the functional analysis. The only file the user needs to edit is the parameters text file where input file paths and parameters are specified.

### Use case data

To demonstrate the application of this workflow for the study of SARS-CoV-2, we applied it to a published transcriptomics dataset. We downloaded raw counts tables from a transcriptomics study of SARS-CoV-2 infected (MOI 2, 24 hour incubation) NHBE cells (Normal Human Bronchial/tracheal Epithelial cell line) with uninfected controls [11] *via* Gene Expression Omnibus (accession GSE147507) [44,45]. SARS-CoV-2 protein–human protein interactions were obtained from Gordon *et al*. [4] *via* IntAct [22,23]. OmniPath and DoRothEA (v2, levels A-C) were downloaded on 15/04/2020. Any genes with log2 fold change ≥ |0.5| and adjusted p value ≤ 0.05 were classed as differentially expressed. All networks were visualised in Cytoscape (v3.7.2).

## Results

### Use case: SARS-CoV-2 infection of lung cells

#### Collection and input of data

To demonstrate the application of this workflow for the study of SARS-CoV-2, we created intracellular signalling networks of NHBE cells (from Normal Human Bronchial/tracheal Epithelial cell lines) upon infection with SARS-CoV-2 based on data published by Blanco Melo *et al*. [11] and viral-human binding protein interactions published by Gordon *et al*. [4].

#### Network reconstruction

**Fig 2:**
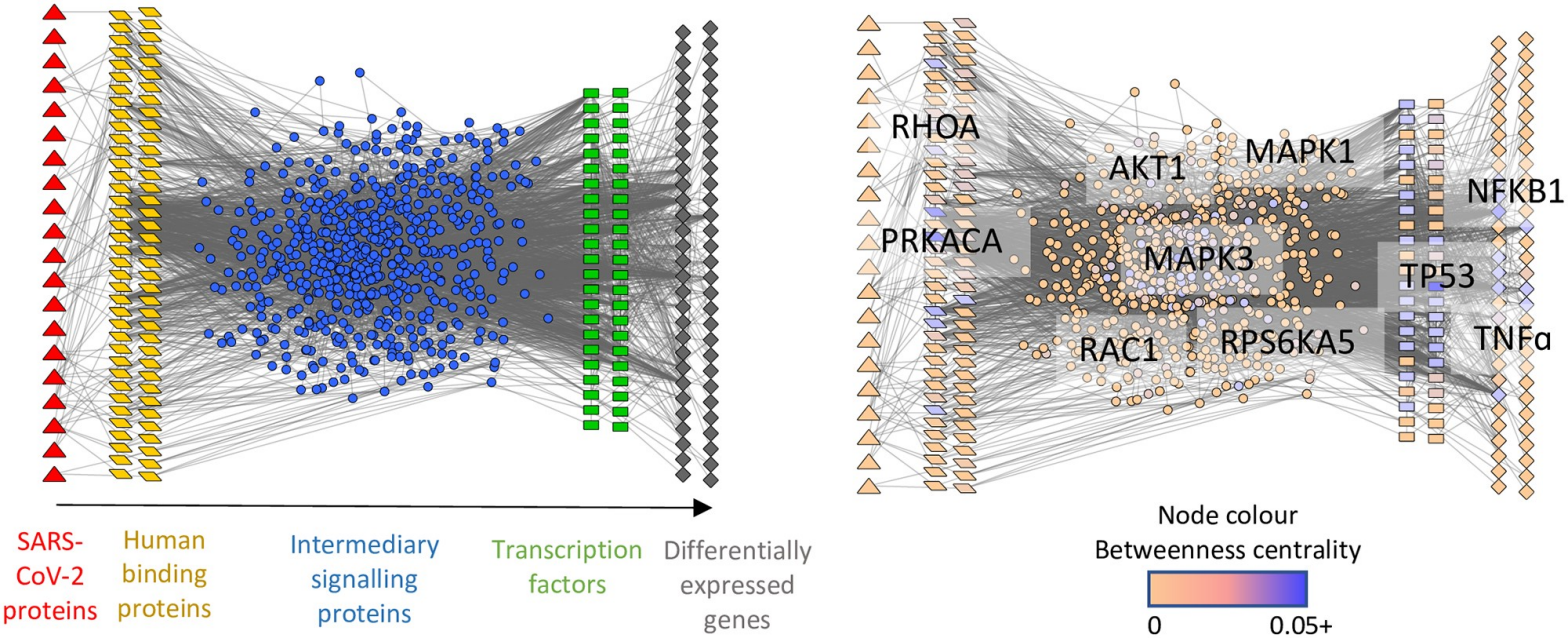
Causal network of SARS-CoV-2-infected NHBE cells. **A)** Signalling flows from left to right: SARS-CoV-2 proteins/protein fragments (red triangles), human binding proteins (yellow parallelograms), intermediary signalling proteins (blue circles), transcription factors (green rectangles) and differentially expressed genes (grey rhombuses). Where a human protein/gene is acting in multiple layers of the network, it is only visualised once based on the following priority: DEGs, binding proteins, TFs, signalling proteins. **B)** Results of betweenness centrality analysis, which measures the global importance of nodes (molecules) in the network. Nodes coloured based on their betweenness centrality parameter, with the gene names of the 10 highest scoring (most central) nodes overlaid. DEGs have log2 fold change ≥ |0.5| and adjusted p value ≤ 0.05.

The resulting causal network contains 804 nodes (molecules) and 5423 interactions (Fig 2A and S1 and S2 Tables and S1 Data).

#### Network investigation

*Centrality measures*. The 10 most central proteins of the reconstructed causal network (based on betweenness centrality) are involved in a wide range of cellular functions (Fig 2B). Taken together these proteins highlight the propensity for SARS-CoV-2 to affect cell proliferation, apoptosis, cell adhesion, exocytosis and proinflammatory immune responses. These functions are influenced through multiple cellular pathways, most notably MAPK/ERK and PI3K/AKT signalling pathways.

*Functional analysis*. Functional overrepresentation analysis identified an enrichment of interleukin and interferon related functions among the network DEGs, in line with previously published findings (S1 Fig and S2 Data) [12,46,47]. However, the primary value of the ViralLink output, in comparison to a basic evaluation of SARS-CoV-2 induced DEGs, lies within the upstream signalling proteins of the reconstructed causal network. Overrepresented functions and pathways of the upstream signalling proteins (human binding proteins, intermediary signalling proteins and TFs) included innate immunity-related functions, platelet signaling, PI3K/AKT signalling, MAPK activation, estrogen receptor-mediated signalling, senescence and a number of growth factor receptor-associated functions (such as VEGF signalling, receptor tyrosine kinases, stem cell growth factor signalling (SCF-KIT) and neurotrophin receptor signaling) (S2 Data and S3 Data). Of these functions, only innate immunity-related functions and MAPK regulation were also identified in overrepresentation analyses within the original publication of the infected NHBE cells [11]–thus highlighting the added benefit of applying ViralLink to DEG lists.

Further, network-aware KEGG pathway analysis identified a number of viral-associated pathways (such as viral carcinogenesis and Epstein-Barr virus infection), indicating a similarity between cellular responses to SARS-CoV-2 and other viruses and validating the context-specificity of the reconstructed causal network (S3 Data). Moreover, network-aware analysis identified overrepresentation of the Reactome function ‘negative regulators of DDX58/IFIH1 signalling’. Importantly, DDX58/IFIH1 signalling results from cellular sensing of cytoplasmic viral nucleic acids and leads to anti-viral innate immune responses such as the production of type I interferon. Many different viruses have been shown to repress this system through varying molecular mechanisms, including hepatitis C and influenza A and B [48]. Of the 11 proteins in the causal network associated with this function, two are theoretically capable of binding to SARS-CoV-2 proteins based on interactions published by [4] (TANK Binding Kinase 1, TBK1 and NLR family member X1, NLRX1). Although further investigation is required, this finding indicates a possible mechanistic explanation for the poor induction of type I interferon response seen in SARS-CoV-2 infections [11,49,50]. Taken together, we show that ViralLink can highlight additional pathways through which SARS-CoV-2 could be affecting the lung epithelial cells, which cannot be identified by looking at the transcriptomic results in isolation.

*Visualisation*. Based on functional overrepresentation analysis, we created a function-specific network by subsetting the causal network. This visualisation was used to further explore the mechanisms of how specific signalling pathways are affecting the DEGs (S2A Fig and S4 Data). Specifically, we generated an innate-immunity associated subnetwork containing all upstream human signalling proteins associated with Reactome functions cytokine signalling in immune system, signaling by interleukins and MyD88-independent TLR4 cascade and all overrepresented functions of the DEGs (in place of the DEG nodes). These pathways contain 9/10 of the top betweenness centrality nodes (all except RHOA), evidencing the centrality and importance of the innate immune response to viral infection. Inspecting the TF layer of this immune subnetwork, we find a number of key TFs including STAT proteins (3 and 4), IRF proteins (1 and 5) and NFKB-related proteins (NFKB1, NFKBIA).

*Cluster analysis*. Finally, we evidenced the application of MCODE clustering analysis using the reconstructed SARS-CoV-2-infected NHBE cell causal network. We identified four clusters containing 15 or more nodes, making up 19% of the network (154/804) (S2B Fig and S2 Table and S1 Data). Assuringly, 9/10 of the top betweenness centrality nodes were included in these four clusters, further confirming the high connectivity and importance of these nodes in the causal network. Functional overrepresentation analysis of the cluster nodes highlighted a functional similarity between all four of the clusters (S2C and S2D Fig and S2 Data). Likely this is due to the high number of inter-cluster molecular interactions and because of the functional similarities between the top central nodes.

Collectively, we show that our systems biology workflow, ViralLink, reconstructs a functionally relevant intracellular signalling network affected by SARS-CoV-2 infection. Investigation of the networks through functional analysis, centrality measures and cluster analysis, combined with network visualisations, enables detailed study of the key proteins and pathways involved in signal transduction.

## Availability and future directions

Infection by SARS-CoV-2 can cause a complex and systemic response by the human body. As such, a better mechanistic understanding of the effects of SARS-CoV-2 will aid identification of effective drug treatments and help to explain the differences in susceptibilities across different populations [51]. This understanding can be gained using cross-disciplinary approaches which combine ‘omics data generation, computational systems biology and validatory web lab experiments [52]. Here we present a computational workflow that can be used to model the cellular response to infection by integrating knowledge of human binding proteins of viral proteins with the transcriptional response of a cell/cell type. Whilst set up primarily to run analyses based on SARS-CoV-2, ViralLink can be applied to any viral infection, provided data is available describing possible interactions between the viral proteins and human proteins.

ViralLink builds on our previously published resource MicrobioLink, which reconstructs networks representing the effect of extracellular and intracellular microbial proteins on cellular processes [21]. Differing from MicrobioLink, ViralLink inputs a predetermined list of viral-host PPIs and focuses only on pathways ending in transcriptional regulation: thereby reducing the complexity of the workflow (for accessibility and speed purposes) and increasing its predictive confidence. Furthermore, ViralLink extends the functionality of MicrobioLink with more advanced network analysis (functional enrichment, clustering and centrality measures) and visualisation options.

By exploiting previously collated and comprehensive collections of molecular interactions [24,25], ViralLink predicts how signal flows from the initial interaction with a viral protein or protein fragment to the ultimate transcriptional changes induced by the virus. Through mapping the direct intracellular effect of viral infection (using a network approach), this workflow enables further investigation into specific signalling pathways and transcription factors which play a key role in signal transduction. Signalling pathways are primarily regulated through post-translational modifications and thus cannot be directly measured using transcriptomics datasets [53]. ViralLink overcomes this problem by predicting signalling using *a priori* molecular interactions, a diffusion algorithm and transcriptomics data. However, this approach is limited by a lack of proteomics or phosphoproteomics data to validate predicted signalling pathways and by possible bias of the *a priori* molecular interactions [54]. Moreover, some of the input genes to ViralLink are often excluded from the output intracellular networks due to a lack of identified upstream pathways. On the other hand, this approach permits identification of differentially regulated genes that are likely affected as a direct result of viral recognition by protein-protein signalling pathways, rather than by secondary signals such as elevated cytokine levels. This permits a more focused analysis of possible drug targets and adds to the understanding of viral pathomechanisms. Finally, the ViralLink workflow employs functional analysis and visualisation methods to aid interpretation of the generated intracellular networks, enabling detailed investigation of key proteins and signalling pathways. Regarding functional analysis, both overrepresentation analysis and a network-aware pathway algorithm called ANUBIX are employed, considering Reactome, Gene Ontology and KEGG annotations [37,55–57]. This varied approach is taken to avoid biases due to variability in results output using different functional analysis tools and functional databases [58].

Due to the modularity of the workflow, it can be easily adjusted or extended. For example, different diffusion and propagation algorithms, such as HotNet2 [59,60], could be implemented as required. The implemented diffusion tool, TieDIE, adds mechanistic value by including only logically coherent paths calculated based on the signs of interactions and activity of source and target nodes. On the other hand, due to requiring signed interactions, this feature results in a reduced possible set of input *a priori* interactions for TieDIE. If desired, a diffusion tool which does not need signed *a priori* interactions can be implemented to increase the input dataset size. Alternatively, a different method, such as an integer linear programming approach which identifies paths based on an optimisation problem (as implemented in CARNIVAL), could be used for network reconstruction [61]. In addition, integration of CARNIVAL could extend the workflow to permit network reconstruction without supplying upstream perturbations (in this case the viral-host protein interactions). Whilst not currently integrated due to data availability issues, the addition of phosphoproteomics data to the pathway propagation methods could improve the prediction of active pathways [62] Alternatively, methods to predict protein activity based on transcriptional signatures, such as VIPER and PROGENy [63,64] could be added to the workflow in addition to network diffusion methods to increase the confidence of pathway predictions. Finally, extension of the network to include additional regulatory molecule types (e.g. miRNAs) or to study non-human hosts, could uncover further mechanisms by which SARS-CoV-2 can affect host cells.

Accessible through GitHub, the workflow requires only the installation of Docker (www.docker.com) enabling user to run ViralLink with only a limited programming ability. At a minimum, only two user specified input files are required: a raw counts table from a transcriptomics study (using gene symbols or UniProt protein IDs) and a two-column metadata table specifying test and control sample IDs. All other files are provided or acquired directly within the workflow—but can be changed by the user if required. However, one limitation of the current workflow is that only basic visualisation is possible programmatically, due to challenges applying one visualisation strategy to all possible output networks, especially with regard to the function-based networks.

In addition to accessibility through a default emphasis on SARS-CoV-2, a key strength of this workflow is the ability to use different input datasets: including different *a priori* molecular interactions, viral-human binding protein interactions and expressed/differentially expressed gene lists. This allows extensive customisation and permits rapid implementation to the most cutting-edge data soon after publication. Running the workflow across different transcriptomics datasets will allow comparison of intracellular viral responses between different cell types, different species and across different conditions (such as severe vs asymptomatic infection). For example, application of the workflow to transcriptomics data from specific immune cell-types, such as macrophages, will likely uncover different host affected signalling pathways and key TFs based on the infected cell-type. This, in turn, could increase our understanding of the role of different immune populations in fighting the infection. In addition, the workflow can be run on data from other SARS-CoV-2 strains when and if they emerge, thereby aiding comparisons of mechanisms of action between the strains.

To evidence the use of this workflow, we applied it to study the effect of SARS-CoV-2 infection in lung epithelial (NHBE) cells using transcriptomics data published by Blanco-Melo *et al*. [11]. In the resulting causal network, DEGs directly affected by SARS-CoV-2 initiated signalling are associated with functions that are known responses to SARS-CoV-2 and other viral infections [65–68]. Upstream of these affected genes we identified a number of potentially important signalling pathways relating to classical viral-immune responses, cell survival and cytoskeletal rearrangements and cell adhesion. On the whole, such pathways and functions were not identified when investigating the DEG lists alone, highlighting the added value of ViralLink [11]. Previous investigation of the first SARS coronavirus (SARS-CoV) identified an inhibition of cell proliferation and an increase in apoptosis regulated to PI3K/AKT signalling [69,70]. Our network of SARS-CoV-2-initiated intracellular signalling suggests that the PI3K/AKT signalling and the AKT1 protein itself are key mediators of SARS-CoV-2 initiated signal transduction and that apoptosis and cell proliferation pathways are affected by SARS-CoV-2, thus highlighting similarities between the two viruses. However, further experimentation and/or data curation is required to confirm the direction of change of specific pathways (up- or downregulated) based on the results of the presented workflow. Together our results indicate that SARS-CoV-2 can affect NHBE cells through a variety of signalling pathways which have been previously associated with similar viruses, including growth factor signalling, MAPK/ERK signalling and PI3K/AKT signalling [67,69–71]. Furthermore, centrality measures and cluster analysis identified proteins which likely play a key role in transduction of these signals and could be good targets for drug treatments.

Several other network reconstruction methods exist which could be and have been applied to study SARS-CoV-2 infections. For example, Messina *et al*. and Gysi *et al*. use diffusion algorithms and other similar methods to investigate proteins in close proximity to human binding proteins based on PPI interactions and gene co-expression networks [16,19,20]. Our workflow builds on these approaches by linking viral proteins to DEGs. Through this method we can observe which signalling pathways mediate the effect of the virus on cellular transcription levels, creating a systems level view of cellular changes as a result of the virus. Using the functional analysis methods and network visualisation capabilities of the workflow, it is possible to predict which viral proteins and host signalling pathways can affect specific cellular functions, enabling more focused identification of drug targets. In addition to protein mediators, this method describes TFs which are involved in the cellular response and identifies which DEGs can be affected as a direct result of viral proteins hijacking host signalling and which are affected through a different mechanism. In addition to the presented workflow, at least one other method has been used to reconstruct SARS-CoV-2-initiated intracellular signalling networks corroborating the benefits of such analysis methods [72]. Differing from the here presented approach, this work uses an extended version of the Signaling Dynamic Regulatory Events Miner method to reconstruct the networks, resulting in a more mathematically complex but computationally heavy analysis [73]. Furthermore, the workflow by Ding *et al*. is a less reusable and accessible workflow because it was designed for a specific analysis.

In conclusion, ViralLink is an easily accessible, reproducible and scalable systems biology workflow to reconstruct and analyse molecular interaction networks representing the effect of the viruses on intracellular signalling. We believe it is the first available integrative workflow for analysing the downstream effects of viral proteins using viral host interactions and host response data. Application of this workflow to study COVID-19 based on a wide variety of conditions and datasets will uncover mechanistic details about SARS-CoV-2 infection of different cell types, providing valuable predictions for wet-lab and clinical validation.

## Supporting information

S1 Fig Overrepresented Reactome functions (A, B) and Gene Ontology Biological Processes (C, D) of the causal network of SARS-CoV-2 infected NHBE cells. A) Top 10 overrepresented Reactome functions of upstream signalling proteins (including human binding proteins, intermediary signalling proteins and TFs) B) Top 10 overrepresented Reactome functions of network DEGs C) Top 10 overrepresented GO-BP functions of upstream signalling proteins (including human binding proteins, intermediary signalling proteins and TFs) D) All overrepresented GO-BP functions of network DEGs (q value ≤ 0.05). DEGs have log2 fold change ≥ |0.5| and adjusted p value ≤ 0.05.
(TIF)

S2 Fig Function-specific network of SARS-CoV-2- infected NHBE cells and cluster analysis on SARS-CoV-2-infected NHBE causal network. **A)** Function-specific subnetwork containing upstream signalling proteins related to the top overrepresented (q value ≤ 0.05) innate immunity-related Reactome functions (cytokine signalling in immune system, signaling by interleukins and MyD88-independent TLR4 cascade) and all overrepresented functions of the DEGs (in place of the DEG nodes). Layers of the network and node shapes same as in Fig 2. DEGs = differentially expressed genes. DEGs have log2 fold change ≥ |0.5| and adjusted p value ≤ 0.05. See S3 Data. **B)** Cluster analysis results where clusters have ≥ 15 nodes. Position of clustered proteins shown within the causal network and to the right as isolated clusters. Nodes coloured by their cluster membership (black = unclustered, green = cluster 1, yellow = cluster 2, pink = cluster 3, blue = cluster 4). Presence of top 10 betweenness centrality nodes in the clusters is indicated to the right of the clusters. **C)** Reactome overrepresentation analysis of the clusters. Top five Reactome terms (by adjusted p value) displayed for each cluster. **D)** Gene Ontology (GO) overrepresentation analysis of the clusters. Top five GO terms (by adjusted p value) displayed for each cluster. See S2 Table and S2 Data.
(TIF)

S1 Table Causal network of SARS-CoV-2-infected NHBE cell. (TXT)

S2 Table Node annotations for causal network of SARS-CoV-2-infected NHBE cell. Includes betweenness centrality measures and clusters identified by MCODE. MCODE clusters 1,3,4 and 5 correspond to the clusters in the manuscript labelled 1,2,3 and 4 respectively. Clusters 2 and 6 were excluded due to size.
(TXT)

S1 Data Causal network of SARS-CoV-2-infected NHBE cell, Cytoscape file. (CYS)

S2 Data Functional overrepresentation results. Reactome and Gene Ontology Biological Processes (q value ≤ 0.05) for differentially expressed genes (DEGs), protein-protein (PPI) interaction nodes (human binding proteins, signalling proteins and transcription factors) and the clusters of the causal network of SARS-CoV-2-infected NHBE cell.
(XLSX)

S3 Data Network-aware functional overrepresentation results. Reactome and KEGG processes (q value ≤ 0.05) for protein-protein (PPI) interaction nodes (human binding proteins, signalling proteins and transcription factors) of the causal network of SARS-CoV-2-infected NHBE cell. Generated using ANUBIX algorithm.
(XLSX)

S4 Data Function-specific network of SARS-CoV-2- infected NHBE cells, Cytoscape file. (CYS)

## References

[1] Teixeira da Silva JA, Tsigaris P, Erfanmanesh M. Publishing volumes in major databases related to Covid-19. Scientometrics. 2020; 1–12. 10.1007/s11192-020-03675-3 32904414 PMC7454548

[2] Barabási A-L, Gulbahce N, Loscalzo J. Network medicine: a network-based approach to human disease. Nat Rev Genet. 2011;12: 56–68. 10.1038/nrg2918 21164525 PMC3140052

[3] Oberfeld B, Achanta A, Carpenter K, Chen P, Gilette NM, Langat P, et al SnapShot: COVID-19. Cell. 2020;181: 954–954.e1. 10.1016/j.cell.2020.04.013 32413300 PMC7190493

[4] Gordon DE, Jang GM, Bouhaddou M, Xu J, Obernier K, White KM, et al A SARS-CoV-2 protein interaction map reveals targets for drug repurposing. Nature. 2020;583: 459–468. 10.1038/s41586-020-2286-9 32353859 PMC7431030

[5] Alto NM, Orth K. Subversion of cell signaling by pathogens. Cold Spring Harb Perspect Biol. 2012;4: a006114 10.1101/cshperspect.a006114 22952390 PMC3428769

[6] Fung S-Y, Yuen K-S, Ye Z-W, Chan C-P, Jin D-Y. A tug-of-war between severe acute respiratory syndrome coronavirus 2 and host antiviral defence: lessons from other pathogenic viruses. Emerg Microbes Infect. 2020;9: 558–570. 10.1080/22221751.2020.1736644 32172672 PMC7103735

[7] Li J, Guo M, Tian X, Wang X, Yang X, Wu P, et al Virus-Host Interactome and Proteomic Survey Reveal Potential Virulence Factors Influencing SARS-CoV-2 Pathogenesis. Med (N Y). 2020; 10.1016/j.medj.2020.07.002 32838362 PMC7373048

[8] Stukalov A, Girault V, Grass V, Bergant V, Karayel O, Urban C, et al Multi-level proteomics reveals host-perturbation strategies of SARS-CoV-2 and SARS-CoV. BioRxiv. 2020; 10.1101/2020.06.17.156455

[9] Lamers MM, Beumer J, van der Vaart J, Knoops K, Puschhof J, Breugem TI, et al SARS-CoV-2 productively infects human gut enterocytes. Science. 2020;369: 50–54. 10.1126/science.abc1669 32358202 PMC7199907

[10] Triana S, Metz Zumaran C, Ramirez C, Kee C, Doldan P, Shahraz M, et al Single-cell analyses reveal SARS-CoV-2 interference with intrinsic immune response in the human gut. BioRxiv. 2020; 10.1101/2020.10.21.348854 PMC8077299 33904651

[11] Blanco-Melo D, Nilsson-Payant BE, Liu W-C, Uhl S, Hoagland D, Møller R, et al Imbalanced Host Response to SARS-CoV-2 Drives Development of COVID-19. Cell. 2020;181: 1036–1045.e9. 10.1016/j.cell.2020.04.026 32416070 PMC7227586

[12] Huang L, Shi Y, Gong B, Jiang L, Liu X, Yang J, et al Blood single cell immune profiling reveals the interferon-MAPK pathway mediated adaptive immune response for COVID-19. medRxiv. 2020; 10.1101/2020.03.15.20033472

[13] Liao M, Liu Y, Yuan J, Wen Y, Xu G, Zhao J, et al Single-cell landscape of bronchoalveolar immune cells in patients with COVID-19. Nat Med. 2020;26: 842–844. 10.1038/s41591-020-0901-9 32398875

[14] Pfaender S, Mar KB, Michailidis E, Kratzel A, Boys IN, V’kovski P, et al LY6E impairs coronavirus fusion and confers immune control of viral disease. Nat Microbiol. 2020;5: 1330–1339. 10.1038/s41564-020-0769-y 32704094 PMC7916999

[15] Guven-Maiorov E, Tsai C-J, Nussinov R. Structural host-microbiota interaction networks. PLoS Comput Biol. 2017;13: e1005579 10.1371/journal.pcbi.1005579 29023448 PMC5638203

[16] Gysi DM, Valle ÍD, Zitnik M, Ameli A, Gan X, Varol O, et al. Network Medicine Framework for Identifying Drug Repurposing Opportunities for COVID-19. arXiv. 2020; 10.1073/pnas.2025581118 PMC8126852 33906951

[17] Zhou Y, Hou Y, Shen J, Huang Y, Martin W, Cheng F. Network-based drug repurposing for novel coronavirus 2019-nCoV/SARS-CoV-2. Cell Discov. 2020;6: 14 10.1038/s41421-020-0153-3 32194980 PMC7073332

[18] Guzzi PH, Mercatelli D, Ceraolo C, Giorgi FM. Master Regulator Analysis of the SARS-CoV-2/Human Interactome. J Clin Med. 2020;9 10.3390/jcm9040982 32244779 PMC7230814

[19] Messina F, Giombini E, Agrati C, Vairo F, Ascoli Bartoli T, Al Moghazi S, et al COVID-19: viral-host interactome analyzed by network based-approach model to study pathogenesis of SARS-CoV-2 infection. J Transl Med. 2020;18: 233 10.1186/s12967-020-02405-w 32522207 PMC7286221

[20] Messina F, Giombini E, Montaldo C, Sharma AA, Piacentini M, Zoccoli A, et al Looking for pathways related to COVID-19 phenotypes: Confirmation of pathogenic mechanisms by SARS-CoV-2—Host interactome. BioRxiv. 2020; 10.1101/2020.11.03.366666 PMC8357963 34385425

[21] Andrighetti T, Bohar B, Lemke N, Sudhakar P, Korcsmaros T. MicrobioLink: An Integrated Computational Pipeline to Infer Functional Effects of Microbiome-Host Interactions. Cells. 2020;9. 10.3390/cells9051278 32455748 PMC7291277

[22] Hermjakob H, Montecchi-Palazzi L, Lewington C, Mudali S, Kerrien S, Orchard S, et al IntAct: an open source molecular interaction database. Nucleic Acids Res. 2004;32: D452–5. 10.1093/nar/gkh052 14681455 PMC308786

[23] Orchard S, Ammari M, Aranda B, Breuza L, Briganti L, Broackes-Carter F, et al The MIntAct project—IntAct as a common curation platform for 11 molecular interaction databases. Nucleic Acids Res. 2014;42: D358–63. 10.1093/nar/gkt1115 24234451 PMC3965093

[24] Türei D, Korcsmáros T, Saez-Rodriguez J. OmniPath: guidelines and gateway for literature-curated signaling pathway resources. Nat Methods. 2016;13: 966–967. 10.1038/nmeth.4077 27898060

[25] Garcia-Alonso L, Holland CH, Ibrahim MM, Turei D, Saez-Rodriguez J. Benchmark and integration of resources for the estimation of human transcription factor activities. Genome Res. 2019;29: 1363–1375. 10.1101/gr.240663.118 31340985 PMC6673718

[26] R Core Team. R: A language and environment for statistical computing. Vienna, Austria: R Foundation for Statistical Computing; 2013.

[27] Love MI, Huber W, Anders S. Moderated estimation of fold change and dispersion for RNA-seq data with DESeq2. Genome Biol. 2014;15: 550–550. 10.1186/s13059-014-0550-8 25516281 PMC4302049

[28] Beal J. Biochemical complexity drives log-normal variation in genetic expression. Engineering Biology. 2017;1: 55–60. 10.1049/enb.2017.0004

[29] Paull EO, Carlin DE, Niepel M, Sorger PK, Haussler D, Stuart JM. Discovering causal pathways linking genomic events to transcriptional states using Tied Diffusion Through Interacting Events (TieDIE). Bioinformatics. 2013;29: 2757–2764. 10.1093/bioinformatics/btt471 23986566 PMC3799471

[30] Consortium UniProt. UniProt: a worldwide hub of protein knowledge. Nucleic Acids Res. 2019;47: D506–D515. 10.1093/nar/gky1049 30395287 PMC6323992

[31] Koschützki D, Schreiber F. Centrality analysis methods for biological networks and their application to gene regulatory networks. Gene Regul Syst Bio. 2008;2: 193–201. 10.4137/grsb.s702 19787083 PMC2733090

[32] Csárdi G, Nepusz T. The igraph software package for complex network research. undefined. 2006;

[33] Ashburner M, Ball CA, Blake JA, Botstein D, Butler H, Cherry JM, et al Gene Ontology: tool for the unification of biology. Nat Genet. 2000;25: 25–29. 10.1038/75556 10802651 PMC3037419

[34] Yu G, Wang L-G, Han Y, He Q-Y. clusterProfiler: an R package for comparing biological themes among gene clusters. OMICS. 2012;16: 284–287. 10.1089/omi.2011.0118 22455463 PMC3339379

[35] Yu G, He Q-Y. ReactomePA: an R/Bioconductor package for reactome pathway analysis and visualization. Mol Biosyst. 2016;12: 477–479. 10.1039/c5mb00663e 26661513

[36] Fabregat A, Jupe S, Matthews L, Sidiropoulos K, Gillespie M, Garapati P, et al The Reactome Pathway Knowledgebase. Nucleic Acids Res. 2018;46: D649–D655. 10.1093/nar/gkx1132 29145629 PMC5753187

[37] Castresana-Aguirre M, Sonnhammer ELL. Pathway-specific model estimation for improved pathway annotation by network crosstalk. Sci Rep. 2020;10: 13585 10.1038/s41598-020-70239-z 32788619 PMC7423893

[38] Pavlopoulos GA, Wegener A-L, Schneider R. A survey of visualization tools for biological network analysis. BioData Min. 2008;1: 12 10.1186/1756-0381-1-12 19040716 PMC2636684

[39] Shannon P, Markiel A, Ozier O, Baliga NS, Wang JT, Ramage D, et al Cytoscape: a software environment for integrated models of biomolecular interaction networks. Genome Res. 2003;13: 2498–2504. 10.1101/gr.1239303 14597658 PMC403769

[40] Su G, Morris JH, Demchak B, Bader GD. Biological network exploration with Cytoscape 3. Curr Protoc Bioinformatics. 2014;47: 8.13.1–24. 10.1002/0471250953.bi0813s47 25199793 PMC4174321

[41] Bader GD, Hogue CWV. An automated method for finding molecular complexes in large protein interaction networks. BMC Bioinformatics. 2003;4: 2 10.1186/1471-2105-4-22 12525261 PMC149346

[42] Brohée S, Faust K, Lima-Mendez G, Vanderstocken G, van Helden J. Network Analysis Tools: from biological networks to clusters and pathways. Nat Protoc. 2008;3: 1616–1629. 10.1038/nprot.2008.100 18802442

[43] Gustavsen JA, Pai S, Isserlin R, Demchak B, Pico AR. RCy3: Network biology using Cytoscape from within R. [version 3; peer review: 3 approved]. F1000Res. 2019;8: 1774 10.12688/f1000research.20887.3 31819800 PMC6880260

[44] Edgar R, Domrachev M, Lash AE. Gene Expression Omnibus: NCBI gene expression and hybridization array data repository. Nucleic Acids Res. 2002;30: 207–210. 10.1093/nar/30.1.207 11752295 PMC99122

[45] Barrett T, Wilhite SE, Ledoux P, Evangelista C, Kim IF, Tomashevsky M, et al NCBI GEO: archive for functional genomics data sets—update. Nucleic Acids Res. 2013;41: D991–5. 10.1093/nar/gks1193 23193258 PMC3531084

[46] Zhang H, Ai J-W, Yang W, Zhou X, He F, Xie S, et al Metatranscriptomic Characterization of COVID-19 Identified A Host Transcriptional Classifier Associated With Immune Signaling. Clin Infect Dis. 2020; 10.1093/cid/ciaa663 32463434 PMC7314197

[47] Chua RL, Lukassen S, Trump S, Hennig BP, Wendisch D, Pott F, et al COVID-19 severity correlates with airway epithelium-immune cell interactions identified by single-cell analysis. Nat Biotechnol. 2020;38: 970–979. 10.1038/s41587-020-0602-4 32591762

[48] Komuro A, Bamming D, Horvath CM. Negative regulation of cytoplasmic RNA-mediated antiviral signaling. Cytokine. 2008;43: 350–358. 10.1016/j.cyto.2008.07.011 18703349 PMC2575845

[49] Sa Ribero M, Jouvenet N, Dreux M, Nisole S. Interplay between SARS-CoV-2 and the type I interferon response. PLoS Pathog. 2020;16: e1008737 10.1371/journal.ppat.1008737 32726355 PMC7390284

[50] Stanifer ML, Kee C, Cortese M, Zumaran CM, Triana S, Mukenhirn M, et al Critical Role of Type III Interferon in Controlling SARS-CoV-2 Infection in Human Intestinal Epithelial Cells. Cell Rep. 2020;32: 107863 10.1016/j.celrep.2020.107863 32610043 PMC7303637

[51] Kirby T. Evidence mounts on the disproportionate effect of COVID-19 on ethnic minorities. Lancet Respir Med. 2020;8: 547–548. 10.1016/S2213-2600(20)30228-9 32401711 PMC7211498

[52] Korcsmaros T, Schneider MV, Superti-Furga G. Next generation of network medicine: interdisciplinary signaling approaches. Integr Biol (Camb). 2017;9: 97–108. 10.1039/c6ib00215c 28106223

[53] Antebi YE, Nandagopal N, Elowitz MB. An operational view of intercellular signaling pathways. Current Opinion in Systems Biology. 2017;1: 16–24. 10.1016/j.coisb.2016.12.003 29104946 PMC5665397

[54] Schaefer MH, Serrano L, Andrade-Navarro MA. Correcting for the study bias associated with protein-protein interaction measurements reveals differences between protein degree distributions from different cancer types. Front Genet. 2015;6: 260 10.3389/fgene.2015.00260 26300911 PMC4523822

[55] Croft D, O’Kelly G, Wu G, Haw R, Gillespie M, Matthews L, et al Reactome: a database of reactions, pathways and biological processes. Nucleic Acids Res. 2011;39: D691–7. 10.1093/nar/gkq1018 21067998 PMC3013646

[56] Kanehisa M, Sato Y, Kawashima M, Furumichi M, Tanabe M. KEGG as a reference resource for gene and protein annotation. Nucleic Acids Res. 2016;44: D457–62. 10.1093/nar/gkv1070 26476454 PMC4702792

[57] The Gene Ontology Consortium. Expansion of the Gene Ontology knowledgebase and resources. Nucleic Acids Res. 2017;45: D331–D338. 10.1093/nar/gkw1108 27899567 PMC5210579

[58] Mubeen S, Hoyt CT, Gemünd A, Hofmann-Apitius M, Fröhlich H, Domingo-Fernández D. The impact of pathway database choice on statistical enrichment analysis and predictive modeling. Front Genet. 2019;10: 1203 10.3389/fgene.2019.01203 31824580 PMC6883970

[59] Leiserson MDM, Vandin F, Wu H-T, Dobson JR, Eldridge JV, Thomas JL, et al Pan-cancer network analysis identifies combinations of rare somatic mutations across pathways and protein complexes. Nat Genet. 2015;47: 106–114. 10.1038/ng.3168 25501392 PMC4444046

[60] Cowen L, Ideker T, Raphael BJ, Sharan R. Network propagation: a universal amplifier of genetic associations. Nat Rev Genet. 2017;18: 551–562. 10.1038/nrg.2017.38 28607512

[61] Liu A, Trairatphisan P, Gjerga E, Didangelos A, Barratt J, Saez-Rodriguez J. From expression footprints to causal pathways: contextualizing large signaling networks with CARNIVAL. NPJ Syst Biol Appl. 2019;5: 40 10.1038/s41540-019-0118-z 31728204 PMC6848167

[62] Dugourd A, Kuppe C, Sciacovelli M, Gjerga E, Emdal KB, Bekker-Jensen DB, et al Causal integration of multi-omics data with prior knowledge to generate mechanistic hypotheses. BioRxiv. 2020; 10.1101/2020.04.23.057893 PMC7838823 33502086

[63] Alvarez MJ, Shen Y, Giorgi FM, Lachmann A, Ding BB, Ye BH, et al Functional characterization of somatic mutations in cancer using network-based inference of protein activity. Nat Genet. 2016;48: 838–847. 10.1038/ng.3593 27322546 PMC5040167

[64] Schubert M, Klinger B, Klünemann M, Sieber A, Uhlitz F, Sauer S, et al Perturbation-response genes reveal signaling footprints in cancer gene expression. Nat Commun. 2018;9: 20 10.1038/s41467-017-02391-6 29295995 PMC5750219

[65] Cao X. COVID-19: immunopathology and its implications for therapy. Nat Rev Immunol. 2020;20: 269–270. 10.1038/s41577-020-0308-3 32273594 PMC7143200

[66] Sallard E, Lescure F-X, Yazdanpanah Y, Mentre F, Peiffer-Smadja N. Type 1 interferons as a potential treatment against COVID-19. Antiviral Res. 2020;178: 104791 10.1016/j.antiviral.2020.104791 32275914 PMC7138382

[67] Arvanitakis L, Geras-Raaka E, Gershengorn MC. Constitutively signaling G-protein-coupled receptors and human disease. Trends Endocrinol Metab. 1998;9: 27–31. 10.1016/s1043-2760(98)00007-1 18406231

[68] Tan M, Liu Y, Zhou R, Deng X, Li F, Liang K, et al Immunopathological characteristics of coronavirus disease 2019 cases in Guangzhou, China. Immunology. 2020;160: 261–268. 10.1111/imm.13223 32460357 PMC7283723

[69] Mizutani T, Fukushi S, Iizuka D, Inanami O, Kuwabara M, Takashima H, et al Inhibition of cell proliferation by SARS-CoV infection in Vero E6 cells. FEMS Immunol Med Microbiol. 2006;46: 236–243. 10.1111/j.1574-695X.2005.00028.x 16487305 PMC7110397

[70] Tsoi H, Li L, Chen ZS, Lau K-F, Tsui SKW, Chan HYE. The SARS-coronavirus membrane protein induces apoptosis via interfering with PDK1-PKB/Akt signalling. Biochem J. 2014;464: 439–447. 10.1042/BJ20131461 25271362

[71] Kumar R, Khandelwal N, Thachamvally R, Tripathi BN, Barua S, Kashyap SK, et al Role of MAPK/MNK1 signaling in virus replication. Virus Res. 2018;253: 48–61. 10.1016/j.virusres.2018.05.028 29864503 PMC7114592

[72] Ding J, Lugo-Martinez J, Yuan Y, Kotton DN, Bar-Joseph Z. Reconstructing SARS-CoV-2 response signaling and regulatory networks. BioRxiv. 2020; 10.1101/2020.06.01.127589 33083801 PMC7574259

[73] Gitter A, Carmi M, Barkai N, Bar-Joseph Z. Linking the signaling cascades and dynamic regulatory networks controlling stress responses. Genome Res. 2013;23: 365–376. 10.1101/gr.138628.112 23064748 PMC3561877

