# Supplementary figures and images for: ViralLink: An integrated workflow to investigate the effect of SARS-CoV-2 on intracellular signalling and regulatory pathways
Source: PLoS Comput Biol. 2021 Feb 3;17(2):e1008685. doi: 10.1371/journal.pcbi.1008685 (PMC7886129; doi:10.1371/journal.pcbi.1008685)

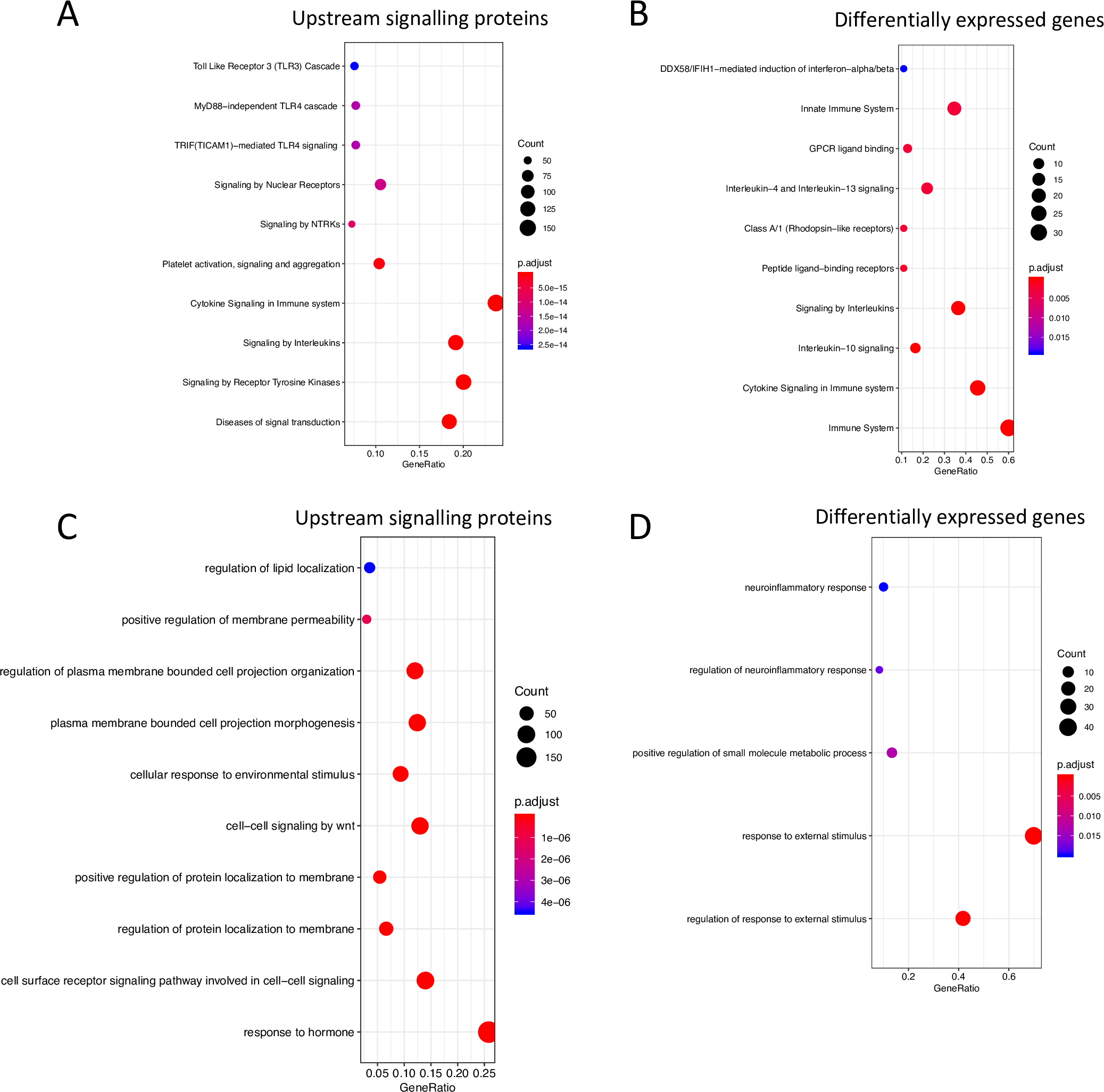

Supplement: S1 Fig — A) Top 10 overrepresented Reactome functions of upstream signalling proteins (including human binding proteins, intermediary signalling proteins and TFs) B) Top 10 overrepresented Reactome functions of network DEGs C) Top 10 overrepresented GO-BP functions of upstream signalling proteins (including human binding proteins, intermediary signalling proteins and TFs) D) All overrepresented GO-BP functions of network DEGs (q value ≤ 0.05). DEGs have log2 fold change ≥ |0.5| and adjusted p value ≤ 0.05. (TIF) [file pcbi.1008685.s001.tif]

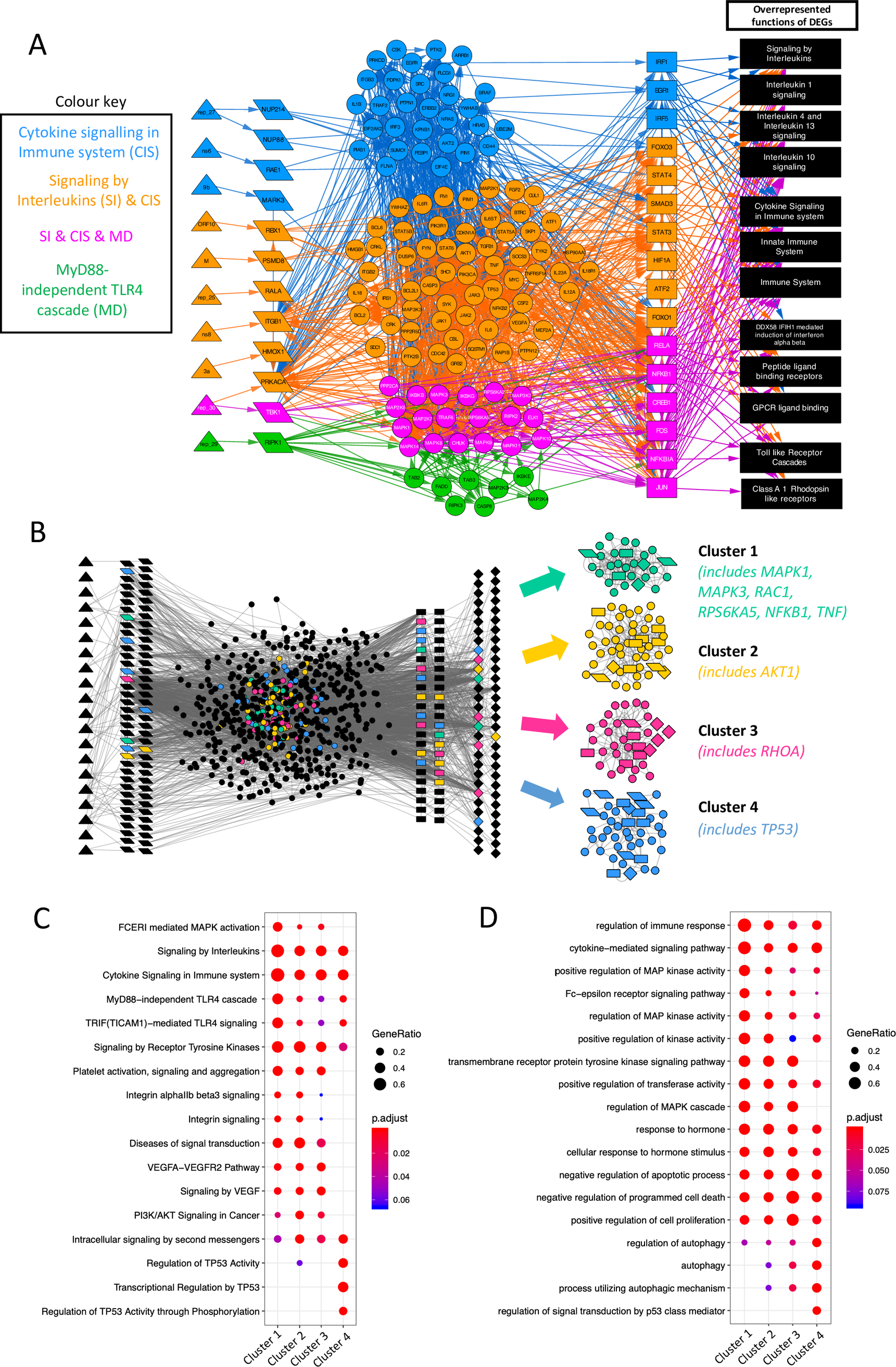

Supplement: S2 Fig — A) Function-specific subnetwork containing upstream signalling proteins related to the top overrepresented (q value ≤ 0.05) innate immunity-related Reactome functions (cytokine signalling in immune system, signaling by interleukins and MyD88-independent TLR4 cascade) and all overrepresented functions of the DEGs (in place of the DEG nodes). Layers of the network and node shapes same as in Fig 2. DEGs = differentially expressed genes. DEGs have log2 fold change ≥ |0.5| and adjusted p value ≤ 0.05. See S3 Data. B) Cluster analysis results where clusters have ≥ 15 nodes. Position of clustered proteins shown within the causal network and to the right as isolated clusters. Nodes coloured by their cluster membership (black = unclustered, green = cluster 1, yellow = cluster 2, pink = cluster 3, blue = cluster 4). Presence of top 10 betweenness centrality nodes in the clusters is indicated to the right of the clusters. C) Reactome overrepresentation analysis of the clusters. Top five Reactome terms (by adjusted p value) displayed for each cluster. D) Gene Ontology (GO) overrepresentation analysis of the clusters. Top five GO terms (by adjusted p value) displayed for each cluster. See S2 Table and S2 Data. (TIF) [file pcbi.1008685.s002.tif]
